# Isolation of Bacteria from Lead-Contaminated Soil and Bacterial Interaction Test with Plant Growing on Lead-Amended Media

**DOI:** 10.21315/tlsr2025.36.2.11

**Published:** 2025-07-31

**Authors:** Dian Siswanto, Nurul Istiqomah, Azizuddin Muhammad Nashafi, Mukhaddam Muhammad, Fathul Mukaromah, Irfan Mustafa

**Affiliations:** 1 Department of Biology, Faculty of Mathematics and Natural Sciences, Brawijaya University, Malang 65145, Indonesia; 2 National Research and Innovation Agency of The Republic of Indonesia (BRIN), Jakarta 10340, Indonesia; 3 Center for Crop Seedling and Plant Protection (BBPPTP) of Surabaya, Jombang 61482, Indonesia

**Keywords:** *Codiaeum variegatum*, *Dracaena reflexa*, *Jasminum humile*, *Lysinibacillus fusiformis*, Plant Growth Promoting Bacteria

## Abstract

The study investigated bacterial isolation from lead-contaminated soil, revealing the lower bacterial density compared to agricultural soil bacteria, indicating soil degradation. Among the isolated bacteria, three isolates with codes L03, L16 and L19 exhibited high tolerance to lead concentrations up to 1,500 mg/L. Selected isolates demonstrated the ability to produce Indole-3-Acetic Acid (IAA) hormone, with one strain notably producing the highest IAA concentration. Furthermore, three isolates exhibited significant lead bioaccumulation efficiency. Molecular identification revealed *Lysinibacillus fusiformis* as the highest IAA-producing strain and lead accumulator. Plant growth experiments analysed the bacteria’s potency to alleviate the heavy metal stress on *Codiaeum variegatum, Dracaena reflexa* and *Jasminum humile*. Additionally, bacterial addition decreased lead absorption only by *D. reflexa*, potentially through biosorption and bioaccumulation mechanisms. Integrating *L. fusiformis* into phytoremediation strategies could offer an effective and sustainable approach for remediating Pb-contaminated environments.

**Highlights**

- Twenty-sixth of Pb-tolerant bacterial isolates from Pb-contaminated soil were identified, with L03, L16 and L19 isolates demonstrating high tolerance to 1,500 mg/L Pb and significant bioaccumulation efficiencies (71.2%–81.1%).
- *Lysinibacillus fusiformis* (L16 isolate) stood out for its ability to produce Indole-3-Acetic Acid (IAA), enhancing plant growth by increasing the fresh weight and root length of *Dracaena reflexa* and reducing Pb concentrations in *Codiaeum variegatum*.
- Integrating IAA-producing Pb-tolerant bacteria, particularly *L. fusiformis*, into phytoremediation strategies was suggested to provide an effective and sustainable solution for remediating Pb-contaminated environments.

## INTRODUCTION

The advent of global industrialisation has significantly exacerbated environmental pollution, particularly through the release of heavy metals into various ecosystems (Bosu *et al.* 2022). Heavy metal contamination, stemming from mining, industrial processes and agricultural activities utilising metal-based compounds, poses a grave threat to environmental and human health (Tangahu *et al.* 2011). Among the plethora of heavy metals, lead (Pb) stands out for its deleterious effects on living organisms, with no known biological or physiological functions and notable toxicity to humans, plants and microorganisms (Genchi *et al.* 2020). Pb, in particular, is associated with a myriad of adverse health effects upon chronic exposure, including high blood pressure, nervous system disorders, renal dysfunction, hyperactivity, anemia and infertility (Yahaghi *et al.* 2018). The accumulation of heavy metals in soil elevates the risk of these contaminants entering the food chain via plant uptake, ultimately posing significant health risks to animals and humans (Uddin *et al.* 2021).

Microbial immobilisation emerges as a promising remediation technique, offering environmental soundness, low production of hazardous materials and minimal energy consumption. Through various mechanisms such as extracellular adsorption, biomineralisation and redox reactions, heavy metal-immobilising bacteria demonstrate the capacity to stabilise toxic metals in soil, thereby mitigating their accumulation in plants (Etesami 2018). Notably, *Agrobacterium tumefaciens* and bacterial consortia, particularly those of *Lysinibacillus* , exhibit significant capabilities in immobilising Cd and Pb, thereby reducing their bioavailability in the soil (El-Meihy *et al.* 2019; Wang *et al.* 2014; Zhang *et al.* 2020b; 2021).

Bacteria contribute to enhancing plant resistance to metal toxicity and decreasing metal accumulation through various mechanisms such as siderophore secretion and modulation of metal availability (Guo *et al.* 2020; Cheng *et al.* 2020; Wang *et al.* 2018). Additionally, Indole-3-Acetic Acid (IAA) plays pivotal roles in plant-microbe interactions and metal stress adaptation. Bacterial IAA production promotes plant growth and enhances nutrient absorption, thereby mitigating metal stress in plants (Mishra *et al*. 2017; Etesami *et al.* 2015). Previous research has shown that the IAA begins to increase the level of protection against adverse external conditions by increasing the coordination of various cellular defense systems (Wahab *et al.* 2022). In general, bacterial IAA can reduce metal stress in plants by increasing plant growth in metal-contaminated soil and increasing nutrient absorption by increasing plant roots (Mishra *et al.* 2017). Bacterial IAA also facilitates adaptation and tolerance to metals in metal-stress plants by inducing physiological changes. This can be observed when IAA is involved in reducing heavy metal toxicity in plants by reducing the absorption and translocation of these heavy metals or stimulating antioxidant enzymes (Etesami *et al.* 2015). Although certain bacterial isolates have been reported to play a role in bioremediation and phytoremediation, exploration and isolation of bacteria from specific Pb-contaminated areas are still needed to develop indigenous bacteria-based mitigation technologies.

This study consists of four stages: (1) isolating bacteria from lead-contaminated soil, (2) testing the obtained bacterial isolates––including analysing the production of IAA hormones and their capacity to accumulate lead—and (3) molecular identification of bacteria. Because several previous studies only stopped at the stage of identifying bacterial isolates, our study sought to evaluate (4) the efficacy of bacterial inoculation on the growth and metal tolerance of *Codiaeum variegatum*, *Dracaena reflexa* and *Jasminum humile* grown in lead-amended media. *C. variegatum* is known for its ability to accumulate and tolerate heavy metals such as lead (Pb). (Herlina *et al*. 2020; Afzal *et al.* 2023). Similarly, *D. reflexa* is easy to grow and effectively accumulates Pb in its roots and stems when exposed to soil contaminated with lead and diesel fuel (Fathia *et al.* 2015; Dadrasnia & Pariatamby 2016; Conte *et al*. 2021). *Jasminum sambac* , commonly known as white jasmine, is another plant recognised for its ability to accumulate lead, with Pb found in its leaves, stems, roots and flowers. *J. sambac* is considered a lead hyperaccumulator (Perveen *et al.* 2011). Given the potential of *J. sambac*, its close relative, *J. humile* (yellow jasmine), presents an intriguing opportunity for further investigation as a potential lead phytoremediation agent. By isolating, selecting and identifying bacteria isolates and elucidating the role of bacteria and plant-microbe interactions in lead-amended media, this research endeavors to contribute to developing sustainable solutions for mitigating heavy metal pollution and thus enhancing food safety.

## MATERIALS AND METHODS

### Soil Sampling and The Soil Chemical Characteristic Measurements

Soil samples were taken from Pb-contaminated soil in Mojokerto Regency, East Java Province, Indonesia, at three different points, which were marked by the codes LKD1, LKD2 and LKD3 with coordinates of 7°22′34.05″ S, 112°27′36.32″ E, 7°22′34.22″ S, 112°27′35.79″ E, 7°22′34.63″S, 112°27′35.15″E, respectively. From each coordinate, a soil sample was taken with three repetitions and then composited. Soil samples were taken at a depth of 0 cm–15 cm from the soil surface using a soil corer (Zhang *et al.* 2020a). Further, the soil samples were put in a plastic bag and then placed in a cool box until they arrived at the laboratory. Soil pH values were measured by pH meter, Pb-soil contents were measured using the Atomic Absorption Spectrophotometer (AAS), and soil organic matter contents were determined using the Walkley and Black method. The basic principle behind the Walkley and Black method is the use of potassium dichromate (K_2_Cr_2_O_2_) as an oxidising agent, which reacts with organic matter (organic carbon) in the soil, converting it into carbon dioxide. The amount of potassium dichromate consumed during the reaction is then measured to estimate the organic carbon content. Since soil organic matter (SOM) is typically considered to contain 58% organic carbon, the organic carbon result is multiplied by a factor of 1.724 to convert from organic carbon to organic matter (Hossain *et al.* 2023).

### Isolation of Bacteria

Bacteria were isolated from Pb-contaminated soil samples using nutrient agar (NA) medium supplemented with Pb. The medium underwent sterilisation at 120°C for 20 min, followed by the addition of filter-sterilised Pb(NO_3_)_2_ solution to achieve a final concentration of 100 mg Pb/L (Kurnia *et al.* 2015). The soil samples were then serially diluted with sterile saline solution (0.85% NaCl). Each 0.1 mL dilution series was inoculated on the media by the spread plate method and incubated at 30°C for four days. Bacterial colonies with different morphological characteristics were selected and purified. The purified bacterial isolates were re-cultured in test tubes with NA media and stored for further research.

### Maximum Pb Concentration Tolerance Test of Bacteria Isolates

The maximum tolerance test was based on the method of De Guzman *et al.* (2016) with slight modification. This method was used to determine the maximum level of lead concentration that the isolates could tolerate. Each purified isolate was grown on the NA media with Pb addition which varies as 100, 300, 600, 1,000 and 1,500 mg Pb/L. The isolates were grown at room temperature for 48 h. Three isolates grown on media containing high concentrations of Pb were selected for use in the next test.

### The Growth Curve of Pb-tolerant Bacteria

The bacteria growth observation was based on the modified Sulistyantini and Utami (2021) method. The starter culture was taken as much as 5 mL, and the optical density (OD) values were equalised between the selected isolates. Then, the culture was added to a sterile culture bottle containing 145 mL of NB medium with a total concentration of 200 mg Pb/L. The bacterial cultures in the bottles were incubated at room temperature on a rotary shaker at a speed of 120 rpm. Bacterial cultures were taken aseptically as much as 5 mL every 1 h for the first 6 , then every 2 h until 31 h, and once at 48 h of incubation. Cultures taken at a predetermined time were measured for absorbance values using a UV-Vis spectrophotometer at 600 nm. The absorbance value obtained was then converted into the number of cells using a linear equation from the standard curve of bacterial isolate cells.

### Bacterial Indole 3-Acetic Acid (IAA) Production Test

Isolates of Pb-tolerant bacteria were cultured in bottles containing 25 mL of sterile nutrient broth (NB) media amended with 0.6 mg/mL L-tryptophan in a rotary shaker at 150 rpm, 30°C. After incubation for 24 h, 48 h and 96 h, 1 mL of the cell-free suspension was mixed with 2 mL of Salkowski’s reagent and allowed to stand at room temperature for 20 min (Yahaghi *et al.* 2018). The pink colour developed was then read at a wavelength of 530 nm. Spectrophotometric absorption values were converted to bacterial IAA concentrations using a pure IAA calibration curve.

#### Pb bioaccumulation test on bacteria isolates

The Pb bioaccumulation test was based on the method of Huang *et al*. (2018) and Shao *et al.* (2019) with modifications. The starter cultures of three selected bacterial isolates that were in the exponential phase were equalised for their absorbance values, then 1 mL was inoculated into a sterile culture bottle containing 30 mL of NB medium and 200 mg/L of Pb. Bacterial cultures were incubated at room temperature for 24 h in a rotary shaker at 150 rpm. Subsequently, it was centrifuged at 3,200 rpm for 10 min, and the supernatant was filtered using filter paper (Whatman No. 42). The Pb content in the supernatant was determined by AAS. Heavy metal bioaccumulation efficiency (R) is calculated as follows:


$$\text{R}=\frac{Co-Cf}{Co}\times 100\%$$

where, R = Heavy metal bioaccumulation efficiency (%), Cf = Final heavy metal concentration (mg/L) and Co = Initial heavy metal concentration (mg/L).

### Bacteria Identification Based on 16S rDNA

Chromosomal DNA of the most potential bacterium was extracted using the Zymo DNA Extraction Kit. The sequence of 16S rDNA was amplified using universal bacterial primers 27f (5′-AGA GTT TGA TCC TGG CTC AG-3′) and 1492r (5′-GGTTACCTTGTTACGACTT-3′). PCR amplification of 16S rDNA was carried out using GoTaq® Green Master Mix in Mastercycler Personal thermocycler (Eppendorf) subjected to initial denaturation at 94°C for 5 min, followed by 35 cycles consisting of denaturation at 94°C for 30 s, annealing at 55°C for 30 s and extension at 72°C for 1.5 min and a final extension of 72°C for 10 min (Rohmah *et al.* 2020). Amplicon of 16S rDNA was purified and sequenced in the 1st BASE, Malaysia. The 16S rDNA sequence of the isolate was aligned with reference strains from GenBank® and analysed using the MEGA 11 programme (Tamura *et al*. 2021). A phylogenetic tree was constructed and inferred with the neighbour-joining algorithm with the Tamura-Nei model using 1000 replicate bootstraps (Saitou & Nei 1987).

### Interaction of Bacteria with Plant Growth on Pb-Amended Media

#### Preparation of the test plants

The test plants used for this study were *C. variegatum* , *D. reflexa* and *J. humile* obtained from the Bumiaji Residents’ Garden in the Bumiaji area, Batu City, East Java Province, Indonesia. The selected plants are plants with a height between 30 cm–45 cm. The planting medium used is a combination of fertile soil, manure, husks and coir pith (Cho-Ruk *et al*. 2006). The planting media were uniform for all treatments; therefore, their physical and chemical properties were not measured. Plants were acclimatised in the experimental garden for seven days before adding lead and bacteria solution. A 200 mL Pb solution containing 100 mg Pb/L was poured on the planting medium surface.

#### Inoculation of bacteria starter

The bacteria starter was prepared by inoculating one loop of bacterial culture on NB media and incubating it at room temperature until the exponential growth phase was reached. Subsequently, the starter was poured into the soil area around the plant (Mello *et al.* 2020). A 100 mL of starter contains 7.4 × 10^8^ bacterial cells/mL.

#### The measurement of growth percentage (fresh weight, plant shoot, plant root), leaf IAA concentration and Pb absorption of plant

The fresh weight, shoot and root of plants were measured 14 days after adding 200 mL of the lead solution containing 100 mg Pb/L to the surface of the growing media, then the increasing percentages were calculated. Control plants grown on media without the addition of bacteria were compared to plants grown on growing media with the addition of bacteria. The level of heavy metal absorption was determined on the whole plant. Root sections were separated and washed extensively with one mM Ca(NO_3_)_2_ ·4H_2_O and then with distilled water to remove Pb ions that were adsorbed on the root surface. The weight of shoots and roots was recorded after drying in an oven at 75°C for 72 h. Oven-dried plant samples were digested in a mixture of concentrated HNO_3_-HCl (70%) and 2H_2_O (30%), and the concentration of Pb in the extract was determined using AAS (Tauqeer *et al*. 2016). Total Pb uptake by plants was calculated as the multiplication of plant dry weight and Pb concentration. In addition, the measurement of the IAA content of plant leaves was carried out using the Salkowski method (Etesami *et al.* 2015).

### Data Analysis

The dataset, including bacterial counts, pH levels, soil organic matter content and Pb concentrations across various locations, was initially presented descriptively without statistical analysis. However, to delve deeper into its insights, statistical scrutiny was applied to two key aspects: bacterial IAA hormone production and the bioaccumulation of Pb by bacteria, which involved employing ANOVA followed by Tukey’s post-hoc analysis to discern significant differences. Furthermore, statistical evaluation was extended to other crucial metrics, including the percentage increases in fresh weight, shoot and root growth of plants, leaf IAA content and plant Pb concentration. Again, ANOVA, followed by Tukey’s post-hoc analysis, was used to identify significant variations across different conditions. In addition to these analyses, principal component analysis (PCA) was conducted to gain insights into the correlation between various parameters in the plant-bacteria interaction test.

## RESULTS

### Soil Bacteria and The Soil Chemical Characteristics

Isolation of bacteria from Pb-contaminated soil samples revealed an average bacterial cell count ranging from 1.1 × 10^4^ CFU/g to 8.5 × 10^4^ CFU/g on NA media with a total Pb concentration of 100 mg/L (Table 1). These bacteria were sourced from soil samples exhibiting pH values spanning from 6.86 to 7.74, organic matter content ranging from 1.06 g/kg to 1.27 g/kg, and Pb concentrations varying between 20.39 mg/kg to 131.01 mg/kg.

### Morphology Colony of Bacteria Isolates and Bacterial Tolerance to Maximum Pb Concentration

Bacteria isolation from soil samples yielded 26 distinct isolates based on their morphological characteristics (Table 2). Subsequently, a maximum concentration tolerance test revealed varying tolerance levels across all isolates. Notably, three isolates, namely L03, L16 and L19, demonstrated remarkable resilience, capable of thriving in Pb concentrations as high as 1,500 mg/L (Table 3).

### Bacterial Growth of Selected Isolates Without and Under Pb Addition

Bacterial growth was observed and presented as a growth curve to determine the growth phases of bacterial isolates and the effect of Pb on bacterial growth. The results were obtained from the growth characteristics of each different bacterial isolate.

**Figure 1:**
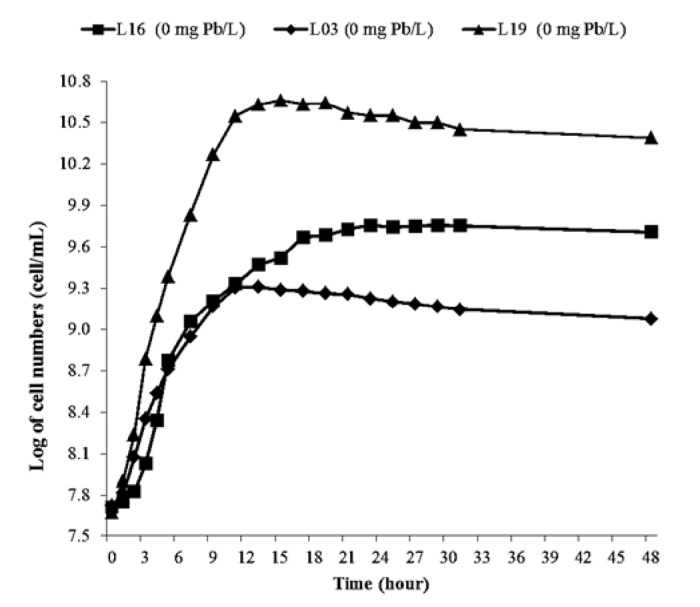
Growth of three selected isolates growth in nutrient broth with 0 mg Pb/L.

**Figure 2:**
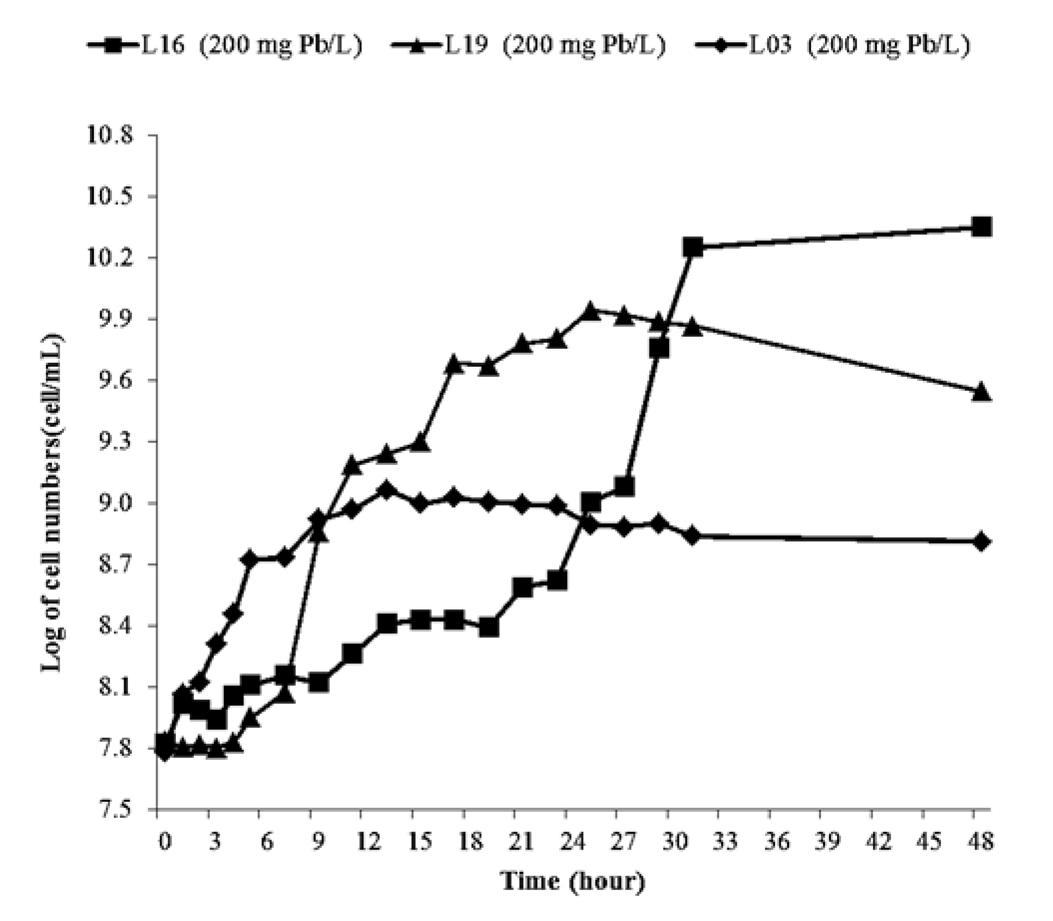
Growth of three selected isolates growth in nutrient broth with 200 mg Pb/L.

Comparative analysis of growth curves for bacterial isolates in control media (Fig. 1) and media supplemented with Pb at 200 mg/L (Fig. 2) revealed a discernible influence of Pb on bacterial growth patterns.

### Bacterial Indole Acetic Acid (IAA) Hormone

**Figure 3:**
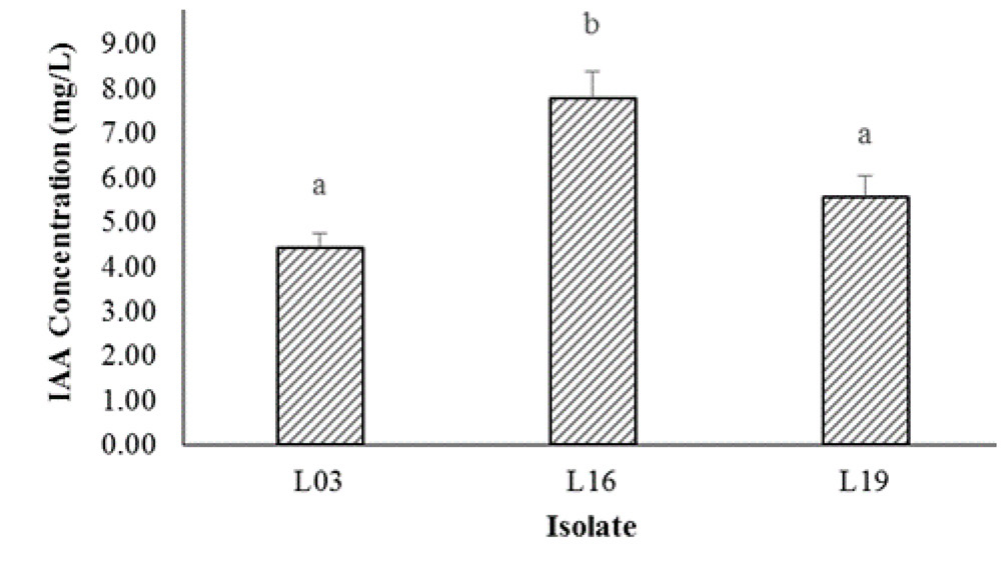
The concentration of IAA production by three selected isolates. Note: Values shown by bars with the same letters are not significantly different (*P* > 0.05).

Various results were obtained based on the IAA production test on three selected isolates with the highest tolerance to Pb. The bacterial IAA hormone produced ranged from 4.42 mg/L to 7.68 mg/L (Fig. 3).

Among the tested isolates, L16 produced the highest concentration of IAA, reaching 7.78 ± 0.61 mg/L, a statistically significant difference compared to the other two isolates (Fig. 3). Conversely, L03 exhibited the lowest IAA concentration at 5.57 ± 0.47 mg/L, while L19 produced IAA at 4.42 ± 0.33 mg/L.

### Bioaccumulation of Pb by Bacteria

**Figure 4:**
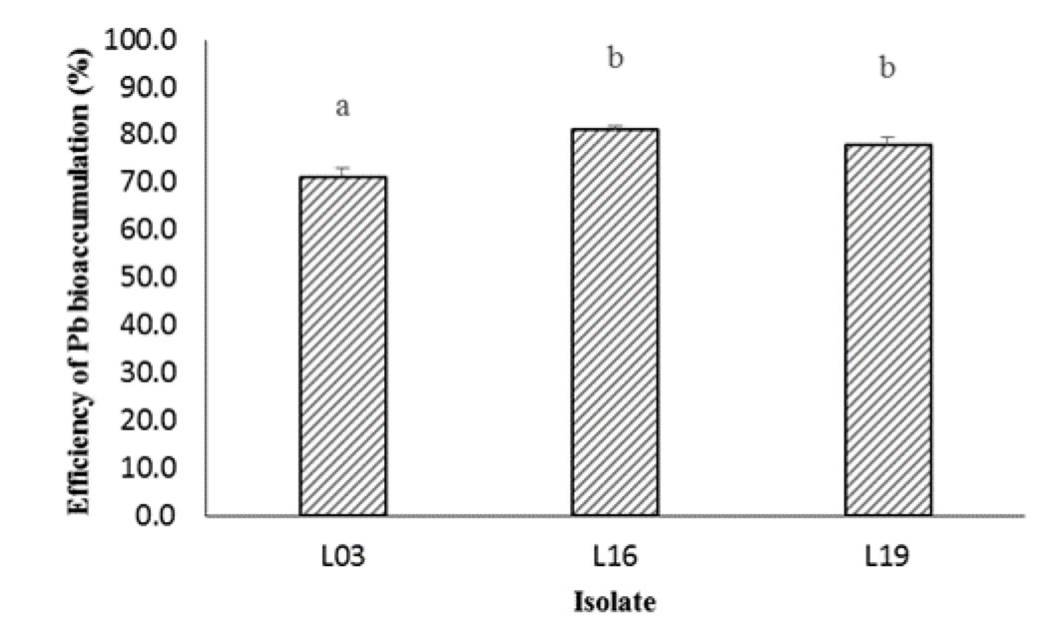
Efficiency of Pb bioaccumulation by three selected isolates. Note: Values shown by bars with the same letters are not significantly different (*P* > 0.05).

Three isolates, specifically L03, L16 and L19, exhibited remarkable potential in accumulating Pb, as demonstrated in Fig. 4. Notably, isolate L03 displayed the Pb bioaccumulation efficiency of 71.2 ± 1.9%, statistically significantly lower than other isolates. The bacteria isolate L16 had a similar Pb bioaccumulation efficiency to the L19 isolate. Each type of bacteria can have a different potential to accumulate heavy metals.

### Molecular Identification of Selected Bacteria Isolate

**Figure 5:**
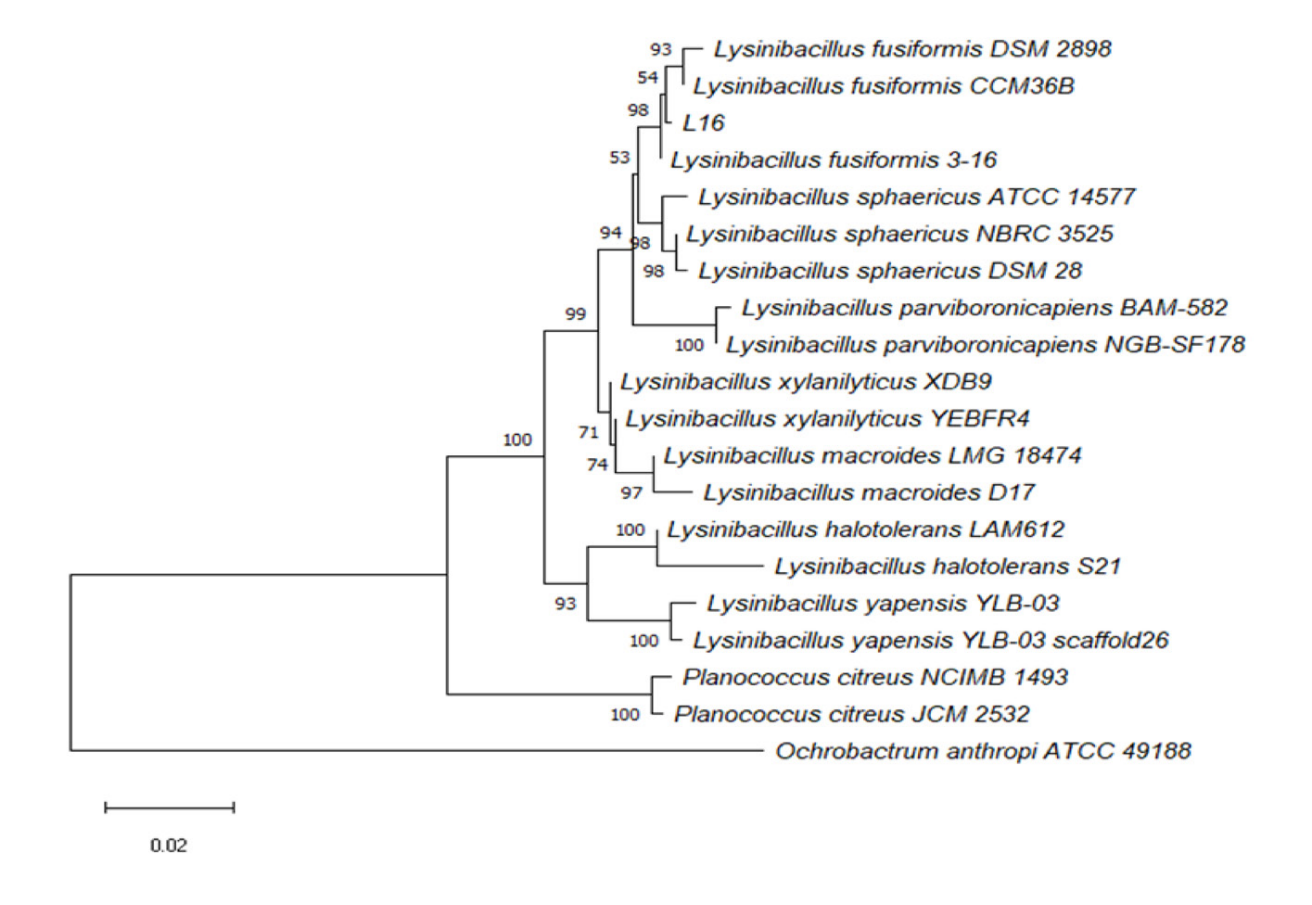
Phylogenic trees of L16 isolate and reference isolates based on the similarity of 16S rDNA sequences with Neighbour-Joining algorithm.

A bacterial phylogeny tree was meticulously constructed for the L16 isolate, which was identified as the most promising bacteria based on the similarity of its 16S rDNA sequences. As depicted in Fig. 5, isolate L16 exhibited a striking 98% similarity to *Lysinibacillus fusiformis* .

### Effect of Pb on Plant Growth

The impact of Pb addition to the growth media on plant development was assessed by measuring plants’ fresh weight, shoot length, and root length after 14 days of treatment, as summarised in Table 4. Notably, plants treated with bacteria tended to have significantly higher values of growth parameters than those without bacterial supplementation, which suggests a positive influence of IAA-producing bacteria addition to plant roots on plant growth, possibly attributable to mechanisms involving Pb immobilisation by bacteria, as evidenced by lower plant Pb and leaf IAA concentrations.

In particular, the percentage increase in fresh weight and root length of *Dracaena reflexa* appeared to be influenced by bacterial-mediated Pb immobilisation, leading to lower plant Pb concentrations. Moreover, the IAA concentration in plant leaves exhibited a noteworthy trend, with plant growth in Pb-amended media (without bacteria addition) showing higher leaf IAA concentrations. This trend was particularly evident when comparing *Codiaeum variegatum* with and without bacterial supplementation.

**Figure 6:**
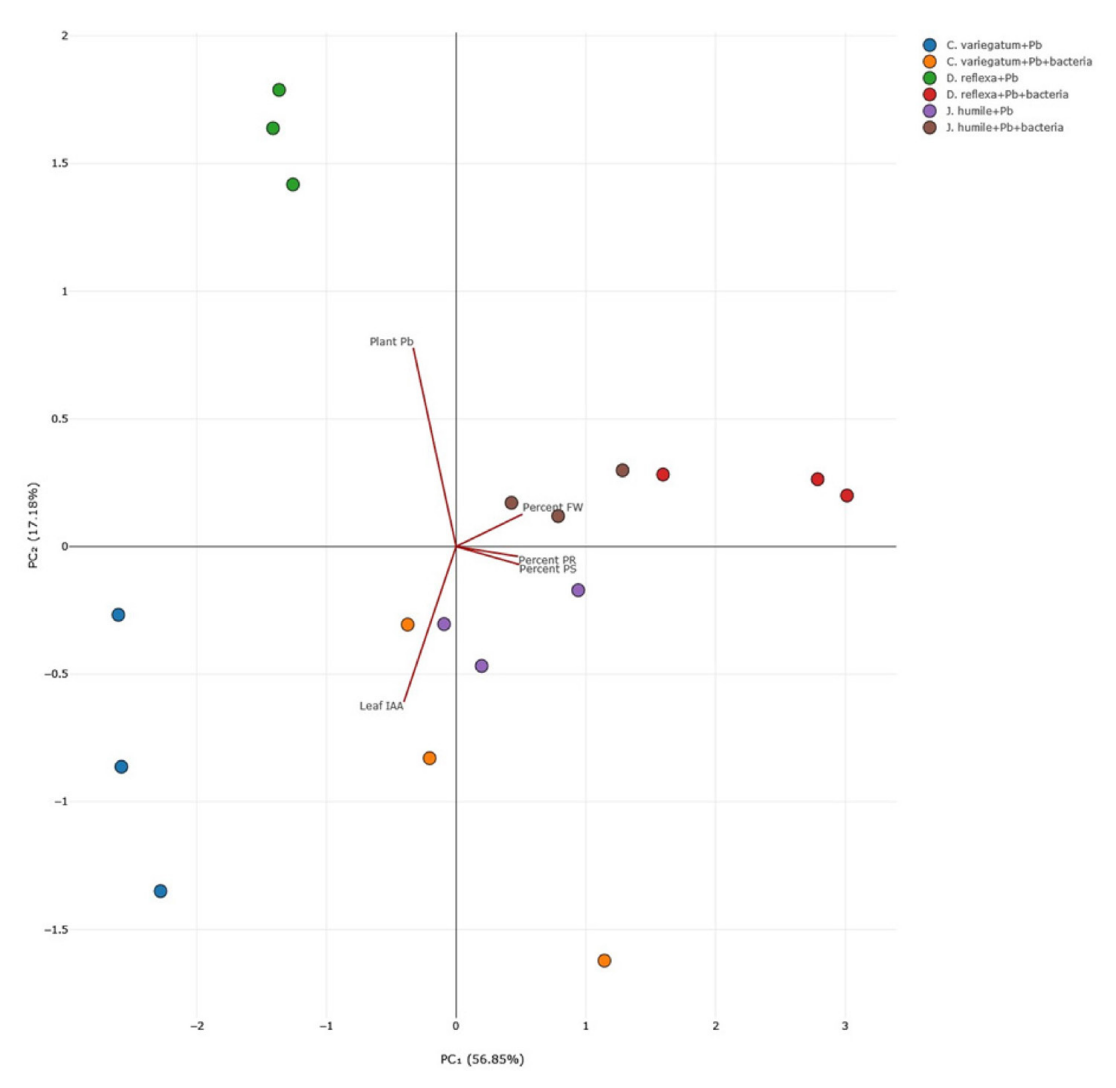
The correlation analysis of parameters by PCA.

Based on PC utilising five parameters—Leaf IAA, Plant Pb, percent FW, Percent PR, and Percent PS (depicted in Fig. 6)—the treatment involving the addition of bacteria to three distinct plant types under Pb conditions yielded four discernible response groups. Group 1 comprises *D. reflexa* + Pb; group 2 encompasses *C. variegatum* + Pb; group 3 includes *C. variegatum* + Pb + bacteria and *J. humile* + Pb; and finally, group 4 consists of *J. humile* + Pb + bacteria and *D. reflexa* + Pb + bacteria.

## DISCUSSION

It is noteworthy that bacterial density in agricultural soil typically falls within the range of 3.0 × 10^6^ to 8.0 × 10^8^ CFU/g (Bhowmik *et al.* 2019; Vieira & Nahas 2005). In contrast, the Pb-tolerant bacterial counts in the soil samples analysed were found to range from 1.1 × 10^4^ to 8.5 × 10^4^ CFU/g. Given that Pb-tolerant bacteria constitute approximately 24.5% of the total bacterial population (Marzan *et al.* 2017), the estimated total bacterial count in our samples would be a maximum of 3.5 × 10^5^ CFU/g. This discrepancy is further underscored by the soil Pb concentration data, suggesting that the soil samples could be characterised as unhealthy soil.

Numerous prior studies have highlighted that bacteria sourced from heavy metal-contaminated soil exhibit heightened metal tolerance compared to those inhabiting regular soil environments (González Henao & Ghneim-Herrera 2021; Jin *et al*. 2018). This resilience is often attributed to the bacteria’s ability to sustain enzymatic activity, enabling growth and adaptation amidst heavy metal stress environments (Seneviratne *et al.* 2016).

According to Wang *et al.* (2015), bacterial growth characteristics are divided into several growth phases, namely the lag or adaptation phase, exponential, stationary and death. Notably, the growth trajectory on Pb-amended media diverged from that on the standard NB medium. Bacterial growth in Pb-containing media predominantly exhibited an adaptation phase during the initial stages. This finding aligns with Arifiyanto *et al.* (2017) assertion that lead toxicity hastens the onset of the death phase while curtailing the stationary phase. Furthermore, insights from Huang *et al.* (2018) underscore the pivotal role of initial heavy metal concentration in modulating microbial growth kinetics under metal-contaminated conditions. Elevated lead concentrations corresponded to heightened growth inhibition, consistent with Marzan *et al*.’s (2017) observations regarding the dose-dependent toxicity of lead. Among the bacterial strains tested, L16 exhibited noteworthy resilience, displaying a logarithmic growth phase from the 3rd to the 31 hour when cultured in NB medium supplemented with an additional 200 mg Pb/L. The phenomena highlights the differential responses of bacterial isolates to Pb-induced stress, emphasising the adaptive capacity of certain strains in hostile environmental conditions.

Under interaction with plants, bacteria utilised IAA to promote plant growth and mitigated abiotic stresses (Etesami & Glick 2024). Bacterial IAA significantly increases plant mineral absorption and nutrient uptake, thereby increasing plant lateral and adventitious root growth (Tirry *et al.* 2018). About 80% of IAA hormone-producing bacteria can generally be found in the soil around plant roots. Bacteria isolated from the soil around plant roots also have the potential to produce IAA hormones even in the presence of heavy metals (Ahemad 2015). The production of IAA by bacteria is subject to various influencing factors, including microbial species, temperature, tryptophan concentration, media pH, agitation and incubation duration (Jasim *et al*. 2014). Heavy metals exert a notable influence on bacterial IAA production. Siderophore-producing bacteria, such as *Escherichia* N16, *Enterobacter* N9 and *Enterobacter* K131, as well as non-siderophore-producing strains like *Serratia* K120 and *Serratia* Mc 119, have demonstrated heightened IAA production in the presence of metals like Cu, Pb, and As in growth media. Specifically, increased bacterial IAA production under Pb exposure is attributed to heightened siderophore production and ACC (1-aminocyclopropane 1-carboxylate) deaminase activity (Carlos *et al.* 2016), which underscores the pivotal role of bacterial phytohormone production, such as IAA, in mediating plant-bacteria interactions and promoting plant growth in heavy metal-contaminated soils, even under stressful conditions (Tirry *et al.* 2018).

Several factors influence the level of heavy metal accumulation by bacteria, including metal concentration, temperature, pH and initial inoculum size. Higher metal concentrations correspond to increased toxicity levels, impacting bacterial growth and accumulation rates. For instance, *Bacillus cereus* R-1 experienced decreased growth and Pb accumulation rates as the Pb concentration in the medium escalated from 10 mg/L to 100 mg/L (Huang *et al.* 2018). Aslam *et al*. (2020) further substantiated the correlation between heavy metal exposure concentration and accumulation levels. *Klebsiella pneumoniae* , *Stenotrophomonas* sp. MB339 and *Staphylococcus* sp. MB371 exhibited varying Pb accumulation percentages at different concentrations, indicating concentration-dependent effects. Despite this, our study’s isolates—L03, L16 and L19—outperformed them at 200 mg/L Pb, showcasing bioaccumulation efficiencies exceeding 70%.

Additionally, incubation temperature plays a crucial role in bacterial heavy metal accumulation. Optimal temperatures ranging from 23°C to 29°C were maintained in our study, facilitating efficient Pb accumulation. The pH of the growth medium also influences bioaccumulation levels, with neutral pH favouring optimal bacterial metabolism and ion transport processes. Notably, our study maintained a pH close to 7, aligning with optimal conditions for heavy metal accumulation. Moreover, the initial inoculum significantly impacts heavy metal accumulation, with higher inoculum sizes correlating with increased absorption areas and enhanced metal ion binding, underscoring bacterial biomass’s importance in facilitating efficient heavy metal uptake (Devika 2013; Shaaban Emara *et al.* 2023).

Based on previous report, the genus *Lysinibacillus*, especially *Lysinibacillus sphaericus* OT4b.31, exhibits notable tolerance to heavy metals, particularly chromium (Cr^6+^) and lead (Pb^2+^). Vegetative cells of this bacterium can withstand Pb^2+^ concentrations up to 1.5 mM, while spore formation occurs at higher concentrations, with spores showing resilience even at concentrations exceeding 20 mM. Notably, the inhibition caused lead is reversible, as the spores can revert to vegetative cells when the metal concentrations are reduced. In bioaccumulation assays, *L. sphaericus* OT4b.31 effectively removed Pb^2+^ from the medium. After 4 h of exposure, the Pb^2+^ concentration decreased by 41 mg/L. Interestingly, the presence of additional metal ions did not affect the bioaccumulation rate, suggesting that the bacterium employs distinct mechanisms to tolerate and resist metal toxicity (Shaw & Dussan 2018).

Further investigation revealed the presence of energy-dependent efflux pumps in *L. sphaericus* OT4b.31. Initially, metal ions such as Pb^2+^ accumulate inside the cells by a protein for divalent cation transport, followed by an active expulsion process to maintain intracellular metal levels below toxic thresholds. The bacterium demonstrated a more rapid response to Pb^2+^ compared to Cr^6+^, with more frequent oscillations in the efflux process when exposed to Pb^2+^. These findings suggest that the efflux pumps play a key role in regulating metal toxicity and maintaining cellular homeostasis, supporting the bacterium’s ability to bioaccumulate metals while simultaneously extruding excess ions (Shaw & Dussan 2018).

Passera *et al*. (2021) elucidated that *L. fusiformis* possesses specific plant growth-promoting attributes, including auxin production and phosphate solubilisation. Additionally, Trivedi *et al.* (2012) reported on the unique interaction profile of *L. fusiformis*, noting its affinity for healthy plants. Remarkably, they observed *L. fusiformis* exclusively in the roots of healthy citrus trees, contrasting with its absence in those afflicted by “*Candidatus Liberibacter asiaticus* ” infection. Generally, Pb exposure poses significant toxicity to plants, with low concentrations stimulating enzymatic activity in soil and microbial biomass, albeit with inhibitory effects observed at higher concentrations. Enzymatic activity, especially that of aryl sulfatase enzymes, tends to decrease with increasing Pb concentrations in plants. However, hyperaccumulator plants exhibit distinct responses to high soil Pb concentrations, demonstrating tolerance and the ability to absorb Pb at elevated levels without compromising metabolic function (Collin *et al*. 2022).

The process of heavy metal absorption by plants through their roots involves several intricate stages. Within the root organs, plants initiate pH adjustments and produce chelating substances known as phytosiderophores. These phytosiderophores bind to metals, facilitating their uptake via active transport mechanisms into root cells. Subsequently, metals are transported through the plant’s vascular system, including the xylem and phloem, to other plant parts such as stems and leaves. This translocation mechanism mitigates metal toxicity within plant cells, representing a crucial detoxification mechanism in plant organs (Banakar *et al.* 2017). The interaction between test plants and bacteria is vital in plant growth and tolerance to toxic compounds (Ma *et al.* 2015). Increased levels of heavy metals in the soil can interfere with phosphate absorption, but bacteria can help plants’ role in P solubilisation (Chen *et al*. 2014). Ma *et al*. (2011) showed that bacteria could affect metal mobility and metal availability by plants through several mechanisms, including soil acidification and phosphate dissolution. The mechanism happens due to the ability of bacteria to assist host plants in adapting to unfavorable environments, as well as to induce plant growth and increase plant metal-stress tolerance (Ma *et al*. 2016).

When considering Pb absorption by plants in the presence of bacteria, reduced absorption levels may result from the intricate interplay between bacteria and plants in the Pb absorption mechanism. Certain genera of soil bacteria are renowned for their capacity to bind heavy metals like Pb. This binding ability not only diminishes the availability of metals for plant uptake but also enhances metal mobility and availability in the soil through mechanisms such as the release of chelating agents, phosphate solubilisation and redox changes (Jing *et al.* 2007; Etesami & Glick 2024).

The selection of *C. variegatum* , *D. reflexa* and *J. humile* in this study was based on their characteristics of tolerance to heavy metals. The plants known as hyperaccumulators have evolved the ability to accumulate and tolerate very high concentrations of heavy metals in their tissues without suffering significant damage. *C. variegatum* was reported for its ability to accumulate and tolerate heavy metals like Pb and studied on its metal tolerance index (MTI), translocation factor (TF) and bioaccumulation factor (BAF), highlighting its potential for remediating lead-contaminated soil (Herlina *et al*. 2020; Afzal *et al*. 2023). Likewise, *D. reflexa* is a hardy plant that effectively accumulates Pb in its roots and stems when grown in Pb- and diesel-contaminated soil, and it can also absorb Pb from motor vehicle exhaust (Fathia *et al*. 2015; Dadrasnia & Pariatamby 2016; Conte *et al.* 2021). Another plant, *Jasminum sambac* (white jasmine), is known for accumulating Pb in its leaves, stems, roots and flowers and is categorised as a lead hyperaccumulator (Perveen *et al*. 2011). Building on the potential of *J. sambac*, its relative, *J. humile* (yellow jasmine), is an interesting candidate for a lead phytoremediation agent.

These heavy metal-tolerant plants possess mechanisms to mitigate the risk of metal poisoning by actively sequestering metals into sub-cellular compartments (Violante *et al*. 2010). Plants accumulate Pb intricately linked to microbial activity, which can alter the bioavailability of heavy metals and facilitate their translocation within plant tissues (Babu *et al.* 2013). Based on PCA, Group 1 correlates strongly with the Plant Pb parameter, while Group 4 exhibits a strong correlation with the percent FW parameter. Groups 2 and 3 also manifest variations in leaf parameters IAA, Percent PR and Percent PS. Integrating Tukey’s post hoc analysis and PCA results underscores *D. reflexa*’s notably most positive response to adding *L. fusiformis*.

## CONCLUSION

This study found 26 Pb-tolerant bacterial isolates from Pb-contaminated soil, with three (L03, L16 and L19) demonstrating high tolerance and survival at 1,500 mg/L Pb. These isolates also showed significant bioaccumulation efficiencies (71.2%–81.1%) and IAA production, suggesting their potential for use in bioremediation. Among them, *L. fusiformis* (L16) emerged as the most promising, as it positively influenced plant growth, notably increasing the fresh weight and root length of *D. reflexa* and reducing Pb concentrations in *C. variegatum* . The results highlight the potential of IAA-producing bacteria in mitigating heavy metal stress, supporting their application in phytoremediation systems. The findings suggest that integrating Pb-tolerant bacteria, particularly *L. fusiformis*, into phytoremediation strategies could offer an effective and sustainable approach for remediating Pb-contaminated environments. Future research should explore the mechanisms underlying bacterial IAA production and its interaction with plants under metal stress to optimise bioremediation processes further. Additionally, using native bacterial communities in conjunction with hyperaccumulator plants could enhance heavy metal removal efficiency, providing a low-cost, eco-friendly alternative to conventional methods. This approach has significant implications for improving soil quality, mitigating industrial pollution and safeguarding human and environmental health.

## Figures and Tables

**Table 1:** Details of sampling location and the bacteria cell number, pH value, organic matter and total Pb of soil in each location.

Sites	Position code	Latitude	Longitude	Bacteria cell number** (CFU/g)	pH value**	Organic matter* (%)	Soil Pb* (mg/kg)
1	LKD1	7° 22′ 34.05″ S	112° 27′ 36.32″ E	1.1 ± 0.5 × 10^4^	7/74 ± 0.29	1.06	131.01
2	LKD2	7° 22′ 34.22″ S	112° 27′ 35.79″ E	8.5 ± 0.3 × 10^4^	6.89 ± 0.31	1.12	29.92
3	LKD3	7° 22′ 34.63″ S	112° 27′ 35.15″ E	8.5 ± 3.0 × 10^4^	7.45 ± 0.18	1.27	20.39

Note:

* : *n* = 1,

** : *n* = 2

**Table 2:** Morphology colony of bacteria isolated from Pb-contaminated soil.

Position code	Isolate code	Morphology colony					

		Shape	Margin	Elevation	Pigmentation	Optical property	Texture
LKD1	L01	Irregular	Curled	Pulvinate	Yellow	Shiny	Contoured
LKD1	L02	Irregular	Curled	Pulvinate	Non pigmented	Shiny	Rough
LKD1	L03	Irregular	Entire	Pulvinate	Brown	Shiny	Rough
LKD1	L04	Irregular	Entire	Raised	Yellow	Shiny	Contoured
LKD1	L05	Irregular	Curled	Convex	Non pigmented	Shiny	Contoured
LKD1	L06	Irregular	Erose	Convex	Yellow	Iridescent	Contoured
LKD2	L07	Rhizoid	Lobate	Flat	Non pigmented	Translucent	Rough
LKD2	L08	Rhizoid	Lobate	Flat	Non pigmented	Shiny	Rough
LKD2	L09	Filamentous	Lobate	Flat	Non pigmented	Translucent	Contoured
LKD2	L10	Filamentous	Lobate	Flat	Non pigmented	Translucent	Rough
LKD2	L11	Irregular	Curled	Convex	Yellow	Iridescent	Concentric (single ring)
LKD2	L12	Irregular	Curled	Convex	Yellow	Shiny	Contoured
LKD2	L13	Irregular	Curled	Raised	Non pigmented	Iridescent	Contoured
LKD2	L14	Circular	Entire	Raised	Yellow	Shiny	Concentric (single ring)
LKD2	L15	Circular	Entire	Pulvinate	Non pigmented	Shiny	Rough
LKD2	L16	Circular	Entire	Pulvinate	Non pigmented	Iridescent	Concentric (single ring)
LKD3	L17	Circular	Entire	Convex	Non pigmented	Shiny	Concentric (single ring)
LKD3	L18	Rhizoid	Lobate	Flat	Non pigmented	Shiny	Rough
LKD3	L19	Irregular	Lobate	Flat	Non pigmented	Shiny	Rough
LKD3	L20	Circular	Entire	Convex	Non pigmented	Shiny	Concentric (single ring)
LKD3	L21	Circular	Entire	Flat	Non pigmented	Shiny	Concentric (single ring)
LKD3	L22	Filamentous	Lobate	Convex	Non pigmented	Iridescent	Contoured
LKD3	L23	Irregular	Entire	Pulvinate	Brown	Shiny	Rough
LKD3	L24	Circular	Entire	Umbonate	Non pigmented	Shiny	Rough
LKD3	L25	Irregular	Entire	Convex	Yellow	Shiny	Contoured
LKD3	L26	Irregular	Entire	Flat	Non pigmented	Shiny	Rough

**Table 3:** Bacteria tolerance test to Pb concentration variations.

Position code	Isolate code	Pb concentration (mg/L)					

		100	300	600	1000	1500	2100
LKD1	L01	+	+	+	−	−	−
LKD1	L02	+	+	−	−	−	−
LKD1	L03	+	+	+	+	+	−
LKD1	L04	+	+	+	+	−	−
LKD1	L05	+	+	+	+	−	−
LKD1	L06	+	+	+	+	−	−
LKD2	L07	+	+	−	−	−	−
LKD2	L08	+	+	+	+	−	−
LKD2	L09	+	+	−	−	−	−
LKD2	L10	+	+	−	c	−	−
LKD2	L11	+	+	+	+	−	−
LKD2	L12	+	+	+	+	−	−
LKD2	L13	+	+	−	−	−	−
LKD2	L14	+	+	+	−	−	−
LKD2	L15	+	+	−	−	−	−
LKD2	L16	+	+	+	+	+	−
LKD3	L17	+	+	−	−	−	−
LKD3	L18	+	+	−	−	−	−
LKD3	L19	+	+	+	+	+	−
LKD3	L20	+	+	+	+	−	−
LKD3	L21	+	+	+	+	−	−
LKD3	L22	+	+	−	−	−	−
LKD3	L23	+	+	+	+	−	−
LKD3	L24	+	+	−	−	−	−
LKD3	L25	+	+	+	+	−	−
LKD3	L26	+	+	−	−	−	−

Note: + = colony appeared, − = colony not appeared.

**Table 4:** The effect of added bacteria on growth, leaf IAA concentrations, Pb concentration of plant growth on Pb amended-media.

No	Plant species	In addition to growth media	Plant growth			Leaf IAA concentration (μg/ml)	Plant Pb concentration (mg/kg)

			Fresh weight (%)	Shoot height (%)	Root length (%)		
1	*C. variegatum*	Pb	4.5 ± 1.5^a^	1.6 ± 0.0^a^	2.9 ± 0.1^a^	37.7 ± 0.6d	0.3 ± 0.1b
2	*C. variegatum*	Pb + bacteria	7.5 ± 0.1a	4.8 ± 2.7a	11.4 ± 0.3^b^	21.7 ± 1.2c	0.2 ± 0.1ab
3	*D. reflexa*	Pb	6.6 ± 1.9^a^	3.1 ± 1.5^a^	4.1 ± 0.1^a^	10.5 ± 3.0b	0.5 ± 0.0c
4	*D. reflexa*	Pb + bacteria	19.8 ± 2.2c	6.1 ± 1.4^a^	16.4 ± 4.4^c^	7.5 ± 2.8ab	0.2 ± 0.0a
5	*J. humile*	Pb	13.3 ± 3.0^b^	3.8 ± 1.2a	2.9 ± 0.1a	8.3 ± 1.8ab	0.1 ± 0.0a
6	*J. humile*	Pb + bacteria	10.1 ± 0.9^ab^	4.5 ± 2.2^a^	11.4 ± 0.3b	5.5 ± 0.5a	0.2 ± 0.0a

Notes: The data is presented as mean ± standard deviation of three individual experiments. Values in the same column with the same letters are not significantly different (*P* > 0.05).
